# The early diversification of influenza A/H1N1pdm

**DOI:** 10.1371/currents.RRN1126

**Published:** 2009-11-12

**Authors:** Martha Nelson, David Spiro, David Wentworth, Jiang Fan, Eric Beck, Kirsten St. George, Elodie Ghedin, Rebecca Halpin, Jayati Bera, Erin Hine, Kathleen Proudfoot, Tim Stockwell, Xudong Lin, Sara Griesemer, Michael Bose, Lisa Jurgens, Swati Kumar, Cecile Viboud, Edward Holmes, Kelly Henrickson

**Affiliations:** ^*^Fogarty International Center, National Institutes of Health, Bethesda, MD; ^†^J. Craig Venter Institute, Rockville, MD, USA; ^‡^Wadsworth Center, NYSDOH and State University of New York; ^§^Medical College of Wisconsin; ^#^Wadsworth Center, New York State Department of Health; ^**^University of Pittsburgh School of Medicine; ^††^JCVI Viral Projects Team Leader; ^‡‡^J.Craig Venter Institute, Rockville, MD, USA; ^¶¶^National Institutes of Health; ^##^J. Craig Venter Institute, Rockville MD; ^***^Influenza virus and Coronavirus Pathogenesis Lab, Wadsworth Center, NYSDOH; ^†††^Laboratory of Viral Diseases, Wadsworth Center, NYSDOH; ^¶¶¶^Fogarty International Center, National Institutes of Health, Bethesda, MD, USA and ^###^The Pennsylvania State University & The Fogarty International Center, NIH

## Abstract

Background Since its initial detection in April 2009, the A/H1N1pdm influenza virus has spread rapidly in humans, with over 5,700 human deaths. However, little is known about the evolutionary dynamics of H1N1pdm and its geographic and temporal diversification.

Methods Phylogenetic analysis was conducted upon the concatenated coding regions of whole-genome sequences from 290 H1N1pdm isolates sampled globally between April 1 – July 9, 2009, including relatively large samples from the US states of Wisconsin and New York.

Results At least 7 phylogenetically distinct viral clades have disseminated globally and co-circulated in localities that experienced multiple introductions of H1N1pdm. The epidemics in New York and Wisconsin were dominated by two different clades, both phylogenetically distinct from the viruses first identified in California and Mexico, suggesting an important role for founder effects in determining local viral population structures.

Conclusions Determining the global diversity of H1N1pdm is central to understanding the evolution and spatial spread of the current pandemic, and to predict its future impact on human populations. Our results indicate that H1N1pdm has already diversified into distinct viral lineages with defined spatial patterns.

## Introduction

In April 2009 a novel influenza A virus ‘H1N1pdm’ was identified in humans, initiating the first influenza pandemic of the 21st century.  As of November 2, 2009, there have been >440,000 laboratory confirmed cases of pandemic H1N1 (H1N1pdm) influenza virus, resulting in > 5,700 deaths worldwide (http://www.who.int/csr/don/2009_10_30/en/index.html, Accessed November 2, 2009) [1].  As most countries have discontinued the reporting of cases, this is thought to represent a minimum of 2-5 million people infected.  H1N1pdm is a novel reassortant between North American and Eurasian swine influenza viruses, containing PB2, PB1, PA, HA, NP, and NS segments from North American triple-reassortant swine viruses and NA and M segments derived from the Eurasian swine lineage [2]. The relatively large genetic distance between H1N1pdm and the most closely related swine influenza viruses suggests that these segments have been circulating undetected for a decade or more, with estimates of the Times to the Most Recent Common Ancestor (TMRCA) for individual genome segments ranging from 9 – 17 years [3].  The relatively low genetic diversity of H1N1pdm suggests that the virus emerged in humans only recently, perhaps in early 2009 [3].      Despite the large-scale surveillance of H1N1pdm at the molecular level, the evolutionary and spatial dynamics of this virus in human populations have yet to be well characterized.  Five early genome variants were identified on the basis of amino acid differences within the United States and Mexico [2], but it is not known whether genetically distinct viral lineages have since proliferated globally, how patterns of genetic diversity vary spatially, or how outbreaks in different localities are linked.  To better understand the evolution, epidemiology, and spatial spread of H1N1pdm during its first months of dissemination in humans, we conducted a large-scale phylogenetic analysis of 290 whole-genome sequences of H1N1pdm sampled globally from April 1 – July 9, 2009.

## Results

### Geographical patterns in viral diversity.  

#### Figure 1

**Figure:**
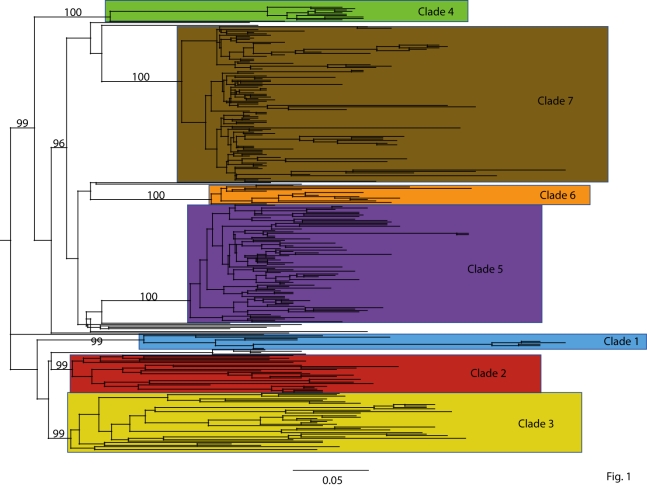
**Fig. 1. **  Maximum Clade Credibility (MCC) tree of the concatenated coding regions of 290 H1N1pdm isolates sampled globally between April 1 – July 9, 2009.  Bayesian posterior probability (BPP) values > 90% are included for key nodes.  The tree is automatically rooted through the assumption of a strict molecular clock, such that tip times represent the time (day) of sampling.  Clades of clearly related isolates are represented by color-coded rectangles.   

To identify discrete genetic lineages of H1N1pdm, we conducted a phylogenetic analysis on 290 concatenated whole-genome sequences of H1N1pdm isolates collected between April 1, 2009 – July 9, 2009, including 81 genomes collected from Wisconsin between April 28, 2009 – May 30, 2009.  The exact collection date was available for each isolate.  Our Bayesian Markov chain Monte Carlo (MCMC) phylogenetic analysis, using the BEAST program [4] (version 1.4.8), revealed 7 discrete clades of H1N1pdm circulating globally (labeled clades 1-7, Fig. 1).  Each clade is defined by long branches and nodes supported by high Bayesian posterior probability (BPP) values (> 90%).  Each clade also is evident on phylogenies inferred using the MrBayes [5] and PAUP* [6] programs (data not shown).  Twenty-one isolates, which include two-thirds (8/12) of all Mexican isolates available for this study, occupy a basal position on the tree with low phylogenetic resolution, and therefore are not members of any of the clades defined here.  Using these basal isolates as a reference point, amino acid changes became fixed in every clade except for clade 3 and in every segment except in the matrix protein (see below, Table 1).  However, none of these amino acid changes are located in sites with known phenotypic effects, such as in antigenic sites on the H1 [7] or those in the NA that are associated with resistance to the neuraminidase inhibitor (NI) antiviral drugs [8] [9]. 

#### Figure 2

**Figure:**
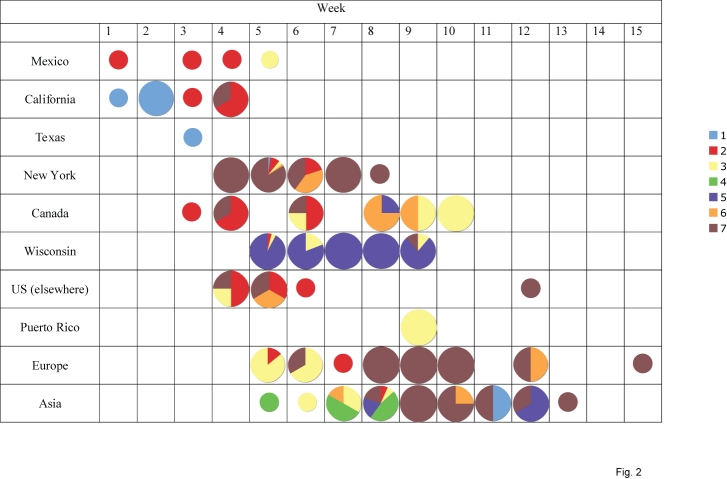
**Fig. 2. **  Changing clade distributions through time and space.  The shading of each circle represents the proportion of isolates belonging to each of the clades identified in Fig. 1 from each locality (y-axis) by epidemic week (x-axis).  The shading of clades is identical to that of Fig. 1.  Week 1 begins with the first isolate collected in this study: April 1, 2009.  Small circles denote weeks for which only a single isolate was available from a locality. 

The 7 clades vary considerably in size: clade 1 contains 9 isolates, clade 4 has 11 isolates, clade 6 has 13 isolates, clade 2 has 25 isolates, clade 3 has 32 isolates, clade 5 has 77 isolates, and clade 7 has 102 isolates.  With the exception of clade 4, which contains isolates collected only from Asia, all clades are geographically dispersed and appear to co-circulate over time and space.  As an extreme case in point, 5 different lineages (clades 1, 2, 3, 6, and 7) all co-circulated in New York during week 5 of this study (April 26 – May 2, 2009, Fig. 2).  Five clades (clades 2, 3, 4, 5, and 7) co-circulated in Asia during week 8, and clades 2, 3, and 5 co-circulated in Wisconsin during week 6.  Although the major epidemics in New York and Wisconsin both were associated with multiple introductions of genetically distinct H1N1pdm, a single clade comprised >80% of virus specimens in each locality: clade 5 in Wisconsin and clade 7 in New York (Fig. 2).  Below we describe the evolutionary dynamics of individual clades in further detail.     Clade 1 includes first H1N1pdm isolates that was identified in our study: A/California/04/2009, collected April 1, 2009.  A Bayesian MCMC analysis estimates that the Time to the Most Recent Common Ancestor (TMRCA) of clade 1 ranges from February 16 – March 16, 2009 (95% highest probability density (HPD)), confirming that clade 1 was one of the earliest to emerge and that it circulated for ~2 – 6 weeks before initial detection (Fig. 3).  However, clade 1 is also the smallest clade in our study (9 isolates) and exhibits only limited geographic dissemination (Fig. 2).  Four unique amino acid changes were observed among the majority (7/9) of clade 1 isolates: S224P in the PA and S91P, A200T, and V323I in the HA (H3 numbering system [10]) (Table 1). 

#### Figure 3

**Figure:**
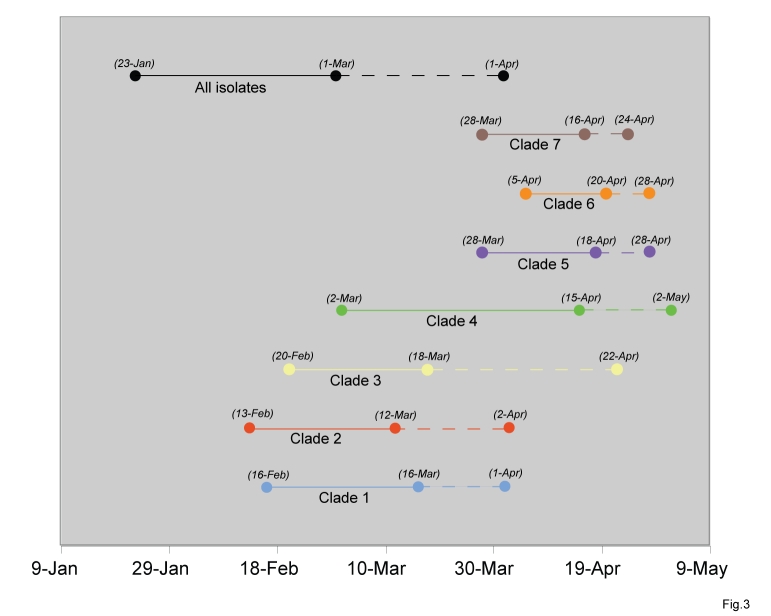
**Fig. 3.**  Bayesian estimates of Times to the Most Recent Ancestor (TMRCA) for each clade identified in Fig. 1.  Points connected by a solid line on the left represent the 95% credible intervals for the TMRCA estimate of each clade, with a dotted line connecting to the date when that clade was first identified in this study.  The shading of points and lines representing clades is identical to Figs. 1 and 2.  

    Clade 2 also contains isolates from among the earliest known cases of H1N1pdm in Mexico and California (e.g., A/Mexico/4108/2009, collected April 2, 2009). The TMRCA of clade 2 suggests it emerged around the same time as clade 1 (February 14 – March 12, 2009, 95% HPD) and circulated for ~3 – 6 weeks prior to detection (Fig. 3).  In contrast to clade 1, clade 2 disseminated widely to Canada, France, Germany, China, and to multiple US states: Indiana, Kansas, Maryland, New York, Washington, and Wisconsin (Fig. 2).  Clade 2 is characterized by two amino acid substitutions: M581L (PA) and T373I (NP) (Table 1).  Following week 8, no isolates collected globally were members of clade 2 (Fig. 2), but further surveillance is needed to determine whether this clade may still be circulating at low levels.     Clade 3 was detected by surveillance several weeks after clades 1 and 2 (A/Arizona/01/2009, collected April 22, 2009).  However, the TMRCA (February 21 – March 19, 2009, 95% HPD) suggests that clade 3 emerged at a similar time as clades 1 and 2, and only was detected later.  Clade 3 is the most geographically diverse, containing isolates from Norway, France, England, Germany, Puerto Rico, China, Japan, Canada, Mexico, and the US states of New York, Wisconsin, and Arizona (Fig. 2).  Although no amino acid changes have been fixed among all clade 3 isolates, at least 13 different amino acid changes were observed among clusters of isolates contained within this clade (data not shown).      Clade 4 is the only clade in this study that contains isolates from only one region: all 11 isolates are from East Asia.  Clade 4 is characterized by fixed amino acid changes in the PB2 (V649I), PB1 (I667T), NP (V100I), and NA (V106I), and NS2 (E63K) (Table 1).  Clade 4 was the last to be detected in this study, as the first isolate (A/Korea/01/2009) was not identified until May 2, 2009.  However, the TMRCA of clade 4 (March 5 – April 18, 2009, 95% HPD) suggests that clade 4 circulated for ~2 – 7 weeks prior to detection, and likely in non-Asian countries as well (Fig. 3).      Clade 5 is the second largest clade identified here, due primarily to the fact that nearly 88% (71/81) of isolates collected from Wisconsin belong to this clade (Fig. 2).  More than 90% (71/77) of isolates in clade 5 were collected from Wisconsin, where the clade was first detected (April 28, 2009) and appears to have proliferated rapidly (Table S1).  Clade 5 also was identified several weeks later in Canada, China, and Japan (Fig. 2).  Although clade 5 was detected <1 week after clade 3, the TMRCA of clade 5 ranges from March 28 – April 18, 2009 (95% HPD), more than one month later than clades 1, 2, and 3 (Fig. 3).  Clade 5 is characterized by amino acid changes in the NP (V100I) and NA (V106I and N248D), relative to basal isolates, but these changes are also observed among clades 6 and 7 (Table 1).       Clade 6 is geographically diverse, given its size in this study (13 isolates) and relatively late TMRCA, ranging from April 4 – April 20, 2009 (95% HPD), which is ~1 – 3 weeks prior to detection (Fig. 3).  Following the collection of the first clade 6 isolates on April 28, 2009 in the US, isolates were collected from Canada, China, Italy, and Japan (Fig. 2).  In addition to the amino acid changes in the NP and NA that also are observed in clade 5, two unique amino acid substitutions in the HA are present among clade 6 isolates: K(-6)E and Q295H, but neither are epitopes (Table 1).      Clade 7 is the largest identified in this study, representing more than one-third of all isolates (35.2%, 102/290) (Table S1).  Almost 83% (67/81) of isolates from New York are members of clade 7.  Clade 7 also contains isolates from Canada, China, Japan, Germany, Italy, Luxembourg, Russia, and the US states of California, Maryland, Massachusetts, Ohio, and Wisconsin (Fig. 2).  Approximately 40% of the isolates collected from Europe and Asia are members of clade 7 (Fig. 2).  Clade 7 is characterized by fixed amino acid changes in the NP (V100I) and NA (V106I and N248D) that are also found in clades 5 and 6, as well as by unique amino acid changes in the HA (S206T) and NS1 (I123V) (Table 1).  Clade 7 was first detected April 24, 2009, and the TMRCA of clade 7 ranges from March 28 – April 16, 2009 (95% HPD), suggesting that clade 7 emerged approximately ~1 – 4 weeks prior to the date of first detection in this study and at a similar time as clade 5 (Fig. 3).        Although none of the 12 HA amino acid substitutions that define clades were located in any of the four antigenic subsites of the H1 (13) (Table 1), amino acid changes of potential antigenic importance were observed among 4/290 isolates in this study.  In the Cb subsite, S79Y and S79F substitutions were identified in the isolates A/Wisconsin/629-D01521/2009 and A/Wisconsin/629-D01705/2009, respectively.  The G159E replacement, located within the Sa subsite, was observed in A/Bayern/62/2009.   And A/Wisconsin/629-D0223/2009 experienced the N160T replacement, located within the Sb subsite. 

#### Table 1    

**Figure:**
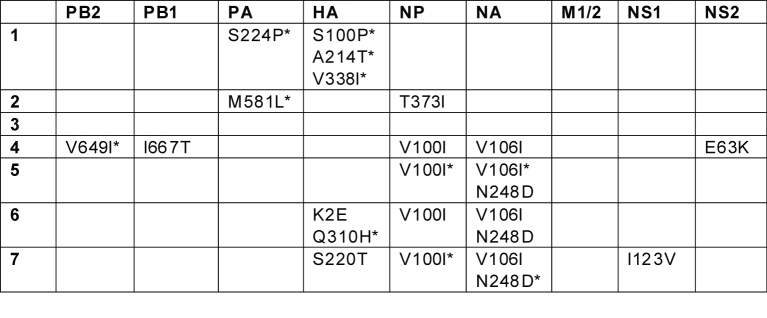

Table 1.  Amino acid changes defining clades 1-7, identified using the MacClade program (30).  The asterisk (*) denotes changes that are present in a majority, but not all of the isolates from a clade.

## Discussion

Our whole-genome phylogenetic analysis of 290 H1N1pdm isolates collected globally, including the intensively sampled populations in Wisconsin and New York, reveals at least 7 major clades of the H1N1pdm influenza virus that have co-circulated over time and space since April 2009.  Clearly, the evolution and spatial-temporal dynamics of the H1N1pdm virus are more complex than the global dissemination of a single viral lineage.  The TMRCA estimates suggest that the 7 major clades emerged between mid-February and late April of 2009, with clades 1, 2, and 3 appearing between mid-February and mid-March, and clades 5, 6, and 7 emerging between mid-March and mid-April. The global origins of clade 4 from Asia remain unclear.  Our estimate for the TMRCA of the entire H1N1pdm viral population ranges from late January – early March, and is consistent with previous estimates [11].  All of these clades circulated for at least two weeks prior to detection in this study, most likely in Mexico and perhaps elsewhere as well.      Due to the co-circulation of these lineages, epidemics in discrete localities involve multiple introductions of genetically distinct H1N1pdm viruses.  However, in locations that are more extensively sampled, such as Wisconsin and New York, a single clade dominated.  It was unexpected that different clades would predominate in Wisconsin and New York, given the similar timing of their outbreaks and presumed population movements, and suggests that founder effects are strong.  Such clear spatial patterns have not been observed previously with seasonal influenza, although sampling has not been as intensive [12].  As viral sequence data becomes available from additional localities, it will be possible to compare the dominant H1N1pdm lineages in other localities and to better understand the spatial movements of the H1N1pdm virus.         It is unclear whether differences in fitness or clinical severity exist among these co-circulating lineages, particularly for clade 7, which was the most commonly detected clade here.  We did not observe any significant differences in age distribution among these clades or any fixed amino acid changes likely to impact fitness [7] [8] [9].  All of these clades appear to transmit successfully in humans, as even the smaller clades were isolated from multiple weeks and in more than one country.  Although fewer cases of H1N1pdm virus have been reported in Asia [1], all 7 clades were detected in at least one Asian country, reflecting the speed and extent of the global dissemination of the H1N1pdm virus.      

## Materials and Methods

 Sample collection and viral isolation in Wisconsin. Patient specimens were collected at Children’s Hospital of Wisconsin (CHW), Dynacare Laboratories (DL), and their associated clinics in Milwaukee, Wisconsin.  Nasopharyngeal were transported in M4 or M6 viral transport medium (Remel, Lenexa, KS) to CHW or DL microbiology laboratories to test for the presence of influenza A, influenza B, or respiratory syncytial virus (RSV) using two multiplex real-time RT-PCR assays.  The CHW test  [13] utilized an in-house assay with automated extraction and real-time PCR amplification and detection.  Influenza A samples were sent to the Midwest Respiratory Virus Program (MRVP) laboratory to distinguish between H1N1pdm and seasonal H1N1 and H3N2 influenza A viruses with real-time RT-PCR or an RT-PCR enzyme hybridization assay [14] [15].  All clinical specimens were de-identified.  H1N1pdm viruses were isolated from clinical specimens by inoculating samples onto Madin-Darby canine kidney (MDCK) cells, grown until 95% confluent, and stored at -80°C.  From a total of ~700 H1N1pdm positive samples collected in Milwaukee beginning April 28, 2009, 90 samples were selected for RNA extraction and genome sequencing: 50 samples were selected from the first week of the outbreak and 10 samples from each of the next four weeks.  The samples were distributed relatively evenly by sex and among three age groups: 0 – 5 years, 5 – 18 years, and 18+ years.

RNA extraction and sequencing of Wisconsin and New York isolates. The extraction of the entire viral RNA genome from isolates from Wisconsin and from clinical samples from New York State was performed at the Wadsworth Center, New York State Department of Health, in Albany, NY, using the NucliSENS® easyMAG™ robot (bioMérieux, Durham, NC), RNeasy Kit (Qiagen, Valencia, CA) or QIAamp Viral RNA Kit with the QIAcube automated extractor (Qiagen).  The influenza A genomic RNA segments were simultaneously amplified from 5 uls of purified RNA using a multisegment RT-PCR strategy [16]. Whole-genome sequencing was performed at the J. Craig Venter Institute (JCVI) in Rockville, MD.  Oligonucleotide primers were designed using a computational PCR primer design pipeline developed at JCVI [17].  Tiled amplicons were designed to have an optimal size of 550 bp with 100 bp overlap in order to provide six fold sequence coverage of the influenza genome.  Influenza segment DNA was amplified using Accuprime Taq at 35 cycles (denaturation: 0.5 min., 94ºC; annealing: 0.5 min., 55ºC; extension: 2 min., 68ºC) and amplicons were treated with Shrimp Alkaline Phosphatase and Exonuclease I.  Sequencing, genome assembly, and closure reactions were performed as described previously [18].  All genome sequences were submitted to GenBank.

Phylogenetic analysis.  The sequence data used in this study came from 81 H1N1pdm isolates collected from Wisconsin between April 28, 2009 – May 30, 2009 and 209 H1N1pdm isolates that were collected globally between April 1, 2009 – July 9, 2009, which were downloaded from the National Center for Biotechnology (NCBI) Influenza Virus Resource (http://www.ncbi.nlm.nih.gov/genomes/FLU/FLU.html) available on GenBank [19].  Only isolates for which whole-genome sequences and exact date of collection were available were used (Table S1).       Nucleotide alignments were manually constructed for the coding regions of the eight genome segments of each isolate using the Se-Al program [20].  To maximize phylogenetic resolution, and given no evidence of genomic reassortment, the eight segments were concatenated.  To infer the evolutionary relationships and timescale for the complete H1N1pdm data set analyzed here, we employed a Bayesian Markov chain Monte Carlo (MCMC) method using a strict molecular clock, as implemented in the BEAST program (version 1.4.8) [4].  Use of a strict molecular clock facilitates computational tractability and is justified here given the strong clock-like evolution of the influenza A virus.  This analysis incorporated a GTR+Γ4 model of nucleotide substitution, with a different substitution rate estimated for each codon position, and a Bayesian skyline coalescent prior, with the latter clearly the best descriptor of the complex population dynamics of influenza virus.  Due to the large size of this data set, we used a UPGMA starting tree and ran a chain length of 500 million (sampling trees every 50,000 generations), until convergence was reached.  The Maximum Clade Credibility (MCC) tree generated by BEAST was analyzed using TreeAnnotator (v1.4.8, available at http://beast.bio.ed.ac.uk) after a 10% burn-in.  Statistical uncertainly is reflected in values of the 95% Highest Probability Density (HPD).  The tree was visualized using FigTree (v1.2.3, available at http://beast.bio.ed.ac.uk), where individual clades supported by high Bayesian posterior probabilities (BPP > 90%) and long branch lengths were identified.       To further assess the reliability of our phylogenetic inferences, two additional trees were estimated (Figs. S2 and S3).  First, a consensus phylogenetic tree was inferred using the Bayesian approach available in MrBayes program [5], employing the GTR+I+Γ4 model of nucleotide substitution, as determined by MODELTEST [21]and run for a total of 5.5 x 106 generations, sampling every 1000.  In contrast to the MCC tree generated by BEAST, this analysis did not assume a strict molecular clock.  Second, we employed the maximum likelihood (ML) method available in the PAUP* package [6].  Because of the very large size of the data set available, this analysis was restricted to Nearest Neighbor-Interchange (NNI) branch-swapping.  In this case a bootstrap resampling process (1,000 replications) using the neighbor-joining (NJ) method was also undertaken, incorporating the ML substitution model.  Both the MrBayes and PAUP* analyses produced phylogenies similar, although less well supported, tree topologies to that obtained using BEAST.     Amino acid changes were identified using the MacClade program [22], with the four antigenic sites (designated Sa, Sb, Ca1/Ca2, and Cb) those identified on the A/PR/8/34(H1N1) virus [7] and converted into H3 numbering [10].  The isolates with the most basal position on the phylogenetic tree (e.g., A/Mexico/4603/2009(H1N1)), not located within any clade, were used as a basis for comparison. 

## Acknowledgements

We would like to thank Drs. Sue Kehl, Nate Ledeboer, Ruoyan Chen, Jessica Trost, Teresa Patitucci, Lorraine Witt, Meredith VanDyke, Elizabeth Davis, and Kate Gaffney from the MRVP and Medical College of Wisconsin, and Gerardo Chowell from Arizona State University for their help in this study.  We are also greatly indebted to all those who submitted H1N1pdm sequences to GenBank’s Influenza Virus Resource.  The Madin-Darby canine kidney (MDCK) cells used for viral isolation were kindly provided by Dr. Xiyang Xu at the Centers for Disease Control and Prevention (Atlanta, GA).  

## Funding Information

This project has been funded in part with federal funds from the National Institute of Allergy and Infectious Diseases (NIAID), National Institutes of Health, Department of Health and Human Services under contract number HHSN272200900007C and by NIAID grants UO1-AI070428, U01-387 AI077988, and U01-AI066584.

## Competing Interests

The authors have declared that no competing interests exist. 
